# Effect of visfatin on K_ATP_ channel upregulation in colonic smooth muscle cells in diabetic colon dysmotility

**DOI:** 10.18632/aging.203871

**Published:** 2022-02-03

**Authors:** Ting Yu, Lin Zhang, Yan Wang, Xiaoxue Shen, Lin Lin, Yurong Tang

**Affiliations:** 1 Department of Gastroenterology, The First Affiliated Hospital with Nanjing Medical University, Nanjing 210029, Jiangsu Province, China; 2 Department of Gastroenterology, Zhongda Hospital, School of Medicine, Southeast University, Nanjing 210029, Jiangsu Province, China

**Keywords:** diabetes, visfatin, GI dysmotility, K_ATP_ channel

## Abstract

The mechanisms of diabetes-related gastrointestinal dysmotility remains unclear. This study aimed to investigate the effect and mechanisms of proinflammatory adipokine visfatin (VF) in the contractile dysfunction of diabetic rat colonic smooth muscle. Twenty Sprague-Dawley rats were randomly divided into control and type 2 diabetes mellitus groups. VF levels in the serum and colonic muscle tissues were tested, the time of the bead ejection and contractility of colonic smooth muscle strips were measured, and the expression of ATP-sensitive potassium (K_ATP_) channels in the colonic muscle tissues was analyzed. *In vitro*, we tested VF’s effects on intracellular reactive oxygen species (ROS) levels, NF-κB’s nuclear transcription, K_ATP_ channel expression, intracellular Ca^2+^ concentrations, and myosin light chain (MLC) phosphorylation in colonic smooth muscle cells (CSMCs). The effects of NAC (ROS inhibitor) and BAY 11-7082 (NF-κB inhibitor) on K_ATP_ expression were also tested. Diabetic rats showed elevated VF levels in serum and colonic muscle tissues, a delayed distal colon ejection response time, weakened contractility of colonic smooth muscle strips, and increased K_ATP_ channel expression in colonic muscle tissues. VF significantly inhibited the contractility of colonic smooth muscle strips from normal rats. In cultured CSMCs, VF caused ROS overload, increased NF-κB nuclear transcription activity and increased expression of Kir6.1, eventually reducing intracellular Ca^2+^ levels and MLC phosphorylation. NAC and BAY 11-7082 inhibited the VF–induced Kir6.1 upregulation. In conclusion, VF may cause contractile dysfunction of CSMCs by upregulating the expression of the Kir6.1 subunit of K_ATP_ channels via the ROS/NF-κB pathway and interfering with Ca^2+^ signaling.

## INTRODUCTION

Diabetes mellitus (DM) and its common complications, such as gastrointestinal (GI) symptoms, have become major worldwide public health problems, with type 2 diabetes (T2DM) making up about 90% of cases [1]. Constipation is the most common GI complaint in patients with T2DM, presenting as infrequent bowel movements, straining, sensation of incomplete evacuation, and abdominal discomfort, with considerable effects on quality of life and healthcare spending [2].

Smooth muscle contractions provide the major driving force for movement of luminal content along the GI tract. Colonic smooth muscle contraction disorders are one of the causes of constipation in patients with T2DM, although other factors including autonomic neuropathy, reduced number of interstitial cells of Cajal networks, loss of enteric neurons, and neuroendocrine imbalances are also involved [3]. The contractile status of colonic smooth muscle cells (CSMCs) is primarily determined by the level of intracellular free Ca^2+^ ([Ca^2+^]_i_), which influxes through voltage-operated Ca^2+^ channels (VOCCs). [Ca^2+^]_i_ can be affected by many factors, such as K^+^ channel activation leading to hyperpolarization and smooth muscle relaxation by inactivating the VOCCs and inhibiting Ca^2+^ entry. The ATP-sensitive potassium (K_ATP_) channel is a hetero-octameric complex, comprising four inwardly rectifying pore-forming Kir6.x and four regulatory sulfonylurea receptor SURx subunits. The increased expression and activity of K_ATP_ channels contribute to decreased smooth muscle motility [4]. Previous studies suggested that K_ATP_ channels (Kir6.1/SUR2B) expression in the vascular smooth muscle of T2DM rats was increased [5], therefore, we speculated that colonic smooth muscle contraction disorders might involve changes in K_ATP_ expression in T2DM rats.

Low-grade systemic inflammation is common in patients with T2DM [6]. The presence of intestinal inflammation might have a negative impact on CSMC contraction [7, 8]. Adipokines mainly derived from adipose tissues, such as leptin and adiponectin, mediate low-level systemic inflammation induced by metabolic disorders, and could play an important role in the occurrence and development of T2DM and its complications [9]. Adipokines regulate the expression or activity of K_ATP_ channels. For example, adipocyte-derived relaxing factor inhibits the contraction of vascular smooth muscle by upregulating K_ATP_ channel expression [10]. Leptin mediates K_ATP_ channel activation in arcuate neurons [11] and increases K_ATP_ channel expression in pancreatic beta-cells [12]. Visfatin (VF), a proinflammatory cytokine, is found in tissues and cells, including adipose tissue, liver, lung, kidney, heart, placenta, muscle cells, and leukocytes [13]. VF levels are elevated in certain pathological states, including T2DM, obesity, and several inflammatory disorders. Increasing VF concentrations were independently and significantly associated with T2DM [14]. Moreover, VF might impair vascular smooth muscle and myometrial contractility [15, 16]. However, the effect of VF on colonic smooth muscle has not been explored. Therefore, the present study aimed to investigate the role of VF in inhibiting colonic contractile activity in T2DM rats and its possible regulation of K_ATP_ channels.

## MATERIALS AND METHODS

### Animals’ models

Male Sprague-Dawley (SD) rats (initial weight = 90–110 g; 4 weeks old), were obtained from the Animal Core Facility of Nanjing Medical University. They were allowed to acclimatize to their surroundings for 1 w and then randomly divided into two experimental groups: Control and T2DM (*n* = 10 per group). T2DM was induced by a single intraperitoneal (i.p.) injection of 0.1 mol/L streptozotocin (STZ, 30 mg/kg body weight) (Sigma, St. Louis, MO, USA) dissolved in citrate buffer after 8 w of high fat-high sugar chow feeding consisted of 67% standard laboratory chow, 2.5% cholesterol, 0.5% sodium cholate, 10% lard and 15% carbohydrate. Control rats fed with standard chow and received equal volumes of buffer by i.p. injection [17]. Ethics approval for all animal experiments was obtained from the Institutional Animal and Use Committee of Nanjing Medical University (Approval ID: NJMU20110587).

### Intraperitoneal glucose tolerance tests

An intraperitoneal glucose tolerance test (IPGTT) was performed at 18 weeks of age. After 6 h of fasting (8:00 to 14:00), rats were weighed, fasting glucose and insulin level was determined from tail vein blood, and then 50% glucose (2 g/kg) was injected into the intraperitoneal cavity, followed by measurement of glucose levels after 30, 60, 90, and 120 min. The IPGTT assay was performed twice in two days. Glucose levels were determined using an AccuChek Compact Plus glucometer (Roche, Basel, Switzerland).

### Serum visfatin assay

Serum visfatin was determined using an enzyme-linked immunosorbent assay (ELISA) kit (E-EL-R1067c; Elabscience Biotechnology Co., Ltd, Wuhan, China) according to the manufacturer's protocol. Briefly, standards and samples were added to wells that were pre-coated with a rat VF-specific antibody; all samples were assayed in duplicate. After 90 minutes of incubation at 37°C, the plate was washed with 1× wash buffer and incubated with biotinylated detection antibody specific for rat VF for 1 h at 37°C. After washing again, an avidin-horseradish peroxidase (HRP)-conjugate was added to each well and incubated for 30 minutes at 37°C. Then, the substrate reagent and stop solution were added to the wells in turn. Finally, absorbance was measured at 450 nm.

### Distal colon ejection response

Rats were anesthetized with diethyl ether, and then a glass bead, about 5 mm in diameter, was inserted into the anus of each rat and pushed into the distal colon to a depth of 3 cm using a plastic rod. The rod was withdrawn slowly so that the glass bead remained in the colon. The time of the bead ejection was defined as the time interval between placing the bead and its discharge.

### Muscle strip contractility assays

The distal colonic tissues (above the pelvic brim) [18] were isolated from control and diabetic rats, and the smooth muscle layer were carefully separated out, washed with Krebs solution (118 mmol/L sodium chloride, 4.7 mmol/L potassium chloride, 1.2 mmol/L magnesium sulphate, 1.2 mmol/L monopotassium phosphate, and 11.1 mmol/L glucose, and the pH was adjusted to 7.2~7.4 with 1M NaOH), and dissected into strips measuring approximately 0.3 × 0.8 cm. The strips were mounted in 15 mL organ baths filled with Krebs solution (37°C, 5% CO_2_/95% O_2_). One end of the strip was fixed to a hook on the bottom of the chamber, while the other end was fixed to an isometric force transducer. All the muscle strips were placed under 1 g of resting tension and allowed to equilibrate for at least 30 min. Then, 10^−4^ mol/L acetylcholine (ACh) (Sigma) was added to induce maximal contractility in each strip. Changes in tension at the transducer were processed through an amplifier and recorded using a dedicated data acquisition system (Alcott-Biotech, Shanghai, China). The mean of the contractile response was determined for muscle strip of each rat treated with or without the K_ATP_ channel blocker, glibenclamide (10 μM). In another experiment, drugs, including the vehicle (phosphate-buffered saline (PBS)) and VF (200 ng/mL), were added sequentially and directly to the Krebs solution when the contractions of the strips from normal SD rats became steady after ACh stimulation; the change of muscle activity was then recorded.

### Immunohistochemical staining

Immunohistochemical staining (IHC) was performed to detect VF, SUR2B, and Kir6.1 expression in the colonic smooth muscle layer of control and diabetic rats. The 4% paraformaldehyde solution-fixed, paraffin-embedded colonic tissue sections were sectioned into 5-μm slices, and imaged using IHC staining using rabbit anti-visfatin polyclonal antibodies (1:500, Abcam, Cambridge, MA, USA), rabbit anti-Kir6.1 polyclonal antibodies (1:200, NOVUS, Vancouver, Canada), and mouse anti-SUR2B monoclonal antibodies (1:500, Sigma). Positive cells appeared brown-yellow, and negative cells were blue-purple. Finally, the number of positive cells was counted using the ‘count small-cells’ function using Image-Pro Plus (version 5.0, Media Cybernetics, Rockville, MD, USA), while area percent and integrated optical density (IOD) percent were measured with the ‘measure stain’ function. Five fields were analyzed on each slide, and the average was taken as the measured value. The scoring was done blind to the tissue source.

### Culture and treatment of colonic smooth muscle cells

Primary colonic smooth muscle cells (CSMCs) were isolated from colon tissues as described previously [19]. Briefly, the serosal and mucosal layers were removed and the remaining muscularis was cut into pieces. After type II collagenase digestion (Sigma-Aldrich, St. Louis, MO, USA), CSMCs were cultured in Dulbecco’s modified Eagle’s medium (DMEM) (Gibco, Grand Island, NY, USA) containing 10% fetal bovine serum (Gibco), 100 U/ml penicillin, and 100 μg/ml streptomycin (Beyotime, Jiangsu, China). Only cells that were more than 95% positive for smooth muscle-specific α-actin were used. CSMCs were incubated with 0, 50, 100, 200, or 300 ng/mL VF for various periods after 24 h of serum starvation. To investigate the potential effect of K_ATP_ channels, CSMCs were transfected with Kir6.1 expression plasmids (Shanghai Genechem Co., Ltd., Shanghai, China) using Lipofectamine 2000 (Invitrogen, Waltham, MA, USA). At 6 h post-transfection and after 12 h of serum starvation, the cells were treated with VF. To further investigate the potential effect of nuclear factor kappa B (NF-κB) and reactive oxygen species (ROS), the NF-κB inhibitor BAY 11-7082 (10 mg/mL) (Beyotime) and the ROS inhibitor N-acetyl-L-cysteine (NAC, 1 mmol/L) (Beyotime) were added separately to the medium 1 h before VF treatment.

### Measurement of intracellular Ca^2+^ concentrations

CSMCs were incubated with 5 μmol/L Fluo-3/AM (Beyotime) for 30 mins at 37°C, washed twice with PBS to remove non-specifically bound dye on the cell surface, and then incubated for a 30 minutes to allow complete deesterification of intracellular AM esters. The cells were resuspended in calcium-free Hank's Balanced Salt Solution (HBSS) base buffer or calcium-containing HBSS buffer (Gibco). Then, the fluorescence intensity (indicating changes in the Ca^2+^ concentration) was measured for 200 seconds with excitation at 488 nm and emission at 515 nm using a confocal laser scanning microscope (LSM510, Zeiss, Wetzlar, Germany). Variations in Ca^2+^ fluorescence intensity were expressed as ratios (F/F_0_) of fluorescence counts (F) relative to baseline values before stimulation (F_0_) [20]. All procedures were carried out in the dark. This experiment was performed three times with different CSMC preparations.

### Measurement of contraction by scanning micrometry

An aliquot of cell suspension containing 10^4^ muscle cells/ml was added to HEPES medium containing the test agents. The reaction was terminated by the addition of Ach (10^−5^ mol/L final concentration). Every 50 isolated cells were counted for use as a control. The length of isolated cells treated with a contractile agent was measured at random. The average length of treated cell group was then compared with the average length of untreated cells group. The contraction was expressed as the percentage decrease in the mean cell length from the control. Percentage decrease was calculated as:


$$\text{Cell contraction (\%)}=\text{100}-\frac{\text{mean cell length of experimental group}}{\text{mean cell length of control groupa}}\times 100$$

### Western blotting

The frozen colonic smooth muscle tissues or cultured cells were homogenized in lysis buffer (Beyotime) to extract protein. The protein concentration was determined using a bicinchoninic acid (BCA) protein assay kit (Beyotime). The protein samples were separated by sodium dodecylsulfate/polyacrylamide gel electrophoresis and transferred onto polyvinylidene fluoride membranes. The membranes were blocked with 5% (w/v) skim milk for 1 h at room temperature, and probed with rabbit anti-myosin light chain (MLC) monoclonal antibodies (1:1000, Cell Signaling Technology, Danvers, MA, USA), rabbit anti-phosphorylated (Ser19) MLC monoclonal antibodies (1:1000, Cell Signaling), mouse anti-SUR2B monoclonal antibodies (1:1000, Sigma), rabbit anti-Kir6.1 polyclonal antibodies (1:500, NOVUS), rabbit anti-phosphorylated IκB-α, and rabbit anti-phosphorylated IκB-α (1:1000, Cell Signaling) at 4°C overnight. Rabbit anti-glyceraldehyde-3-phosphate dehydrogenase (GAPDH) monoclonal antibodies (1:1000, Beyotime) were used as an internal reference. After washing three times with Tris-buffered saline and Tween 20 (TBST) for 5 minutes, the membranes were probed with corresponding HRP-conjugated secondary antibodies (Beyotime) for 1 h at room temperature. Immunoreactive Protein bands were detected using enhanced chemiluminescence western blotting reagents (Thermo Fisher Scientific, Waltham, MA, USA) and analyzed using Image J (NIH, Bethesda, MD, USA).

### Quantitative real-time reverse transcription polymerase chain reaction

Total RNA was isolated from the CSMCs or liquid nitrogen-frozen colon tissue using the TRIzol reagent (Invitrogen) and quantified using a Nanodrop 2000 Spectrophotometer (Nanodrop Technologies, Wilmington, DE, USA). After reverse transcription, quantitative real-time polymerase chain reaction (qPCR) was performed using SYBR Premix Ex Taq (Takara, Shiga, Japan) according to the manufacturer’s instructions. The primer sequences were as follows: Kir6.1 forward, 5′-TGCTCTTCGCTATCATGT-3′, reverse, 5′-GTTTTCTTGACCACCTGGAT-3′; SUR2B forward, 5′-ATGAAGCCACTGCTTCCATC-3′, reverse, 5′-ATCCGTCAAAGTTGGCAAAG-3′; GAPDH forward, 5′-GGCCTTCCGTGTTCCTACC-3′, reverse, 5′-CGCCTGCTTCACCACCTTC-3′. GAPDH was used as an internal reference. The relative level of mRNA was quantified using the 2^−ΔΔCt^ method. Each experiment was performed in triplicate.

### NF-κB activity assay

NF-κB activity was evaluated using an NF-κB Activation-Nuclear Translocation Assay Kit (Beyotime) according to the manufacturer’s instructions. Briefly, CSMCs cultured in cover glass bottom dishes were fixed using staining blocking buffer, incubated with anti-NF-κB/P65 antibodies at 4°C overnight, incubated with Cy3-labeled goat anti-rabbit fluorescent antibodies at room temperature for 1 h, and then stained with 4′,6-diamidino-2-phenylindole (DAPI) for 5 mins. The cells were visualized using a confocal laser scanning microscope (LSM510, Zeiss). Besides, phosphorylation of IκB-α by western blot analysis was also used to evaluate the activity of NF-κB.

### Flow cytometric measurement of ROS

ROS were measured using a ROS assay kit (Beyotime). Treated CSMCs were loaded with 10 μM 2,7-Dichlorodi-hydrofluorescein diacetate (DCFH-DA) for 20 minutes at 37°C, and then washed with serum-free DMEM three times. The cells were resuspended in PBS. All the operations were carried out in the dark until flow cytometry analysis. Measurement of ROS formation was performed using flow cytometry with a 488 nm excitation beam and a 515 nm emission beam.

### Statistical analysis

SPSS v14.0 (IBM Corp., Armonk, NY, USA) was used for statistical analysis. All results are expressed as the mean ± standard deviation (SD). The differences between the treatment groups were analyzed using Student’s *t* test or one-way analysis of variance (ANOVA). Statistical significance was accepted at *P* < 0.05.

## RESULTS

### Evaluation of the animal model

**Figure 1:**
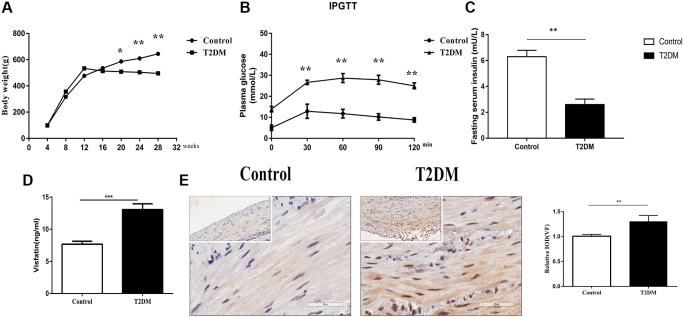
**Evaluation of the animal model.** (**A**) Body weight following an injection of STZ in the control and treatment groups. (**B**) Glucose tolerance test results in the two groups at 18 w. (**C**) Fasting serum insulin level in the two groups at 18 w. (**D**) Serum VF levels in the two groups at 18 w. (**E**) Immunohistochemical staining of VF in the colon muscle layers in the two groups at 8 w. Left panel: Representative image; right panel: Quantitative analysis. Low magnification, 200×, scale bar = 100 μm; high magnification, 400×, scale bar = 50 μm. ^*^P < 0.05, ^**^P < 0.01, ^***^P < 0.001, control group *n* = 10, T2DM group *n* = 8.

One week after modeling, the eight rats in the T2DM group had blood glucose levels >16.7 mmol/L, and were considered diabetic and suitable for further research. The body weights of the STZ-induced rats (T2DM group *n* = 8) were lower than those of the controls (*n* = 10) (Figure 1A). The fasting blood glucose values of the T2DM group were significantly higher than those of the controls. After stimulation with 50% glucose, the peak blood glucose exceeded normal, and the peak was delayed (Figure 1B), indicating that the T2DM rats were established successfully. The fasting serum insulin level was lower in the diabetic T2DM group (*P* < 0.01, Figure 1C). Compared with that of the controls, the serum VF concentrations were higher in the T2DM rats (*P* < 0.001, Figure 1D). IHC staining showed a statistically significant increase in the VF levels in the colonic smooth muscle layers obtained from T2DM rats (*P* < 0.01, Figure 1E).

### Colonic motility dysfunction of T2DM rats

**Figure 2:**
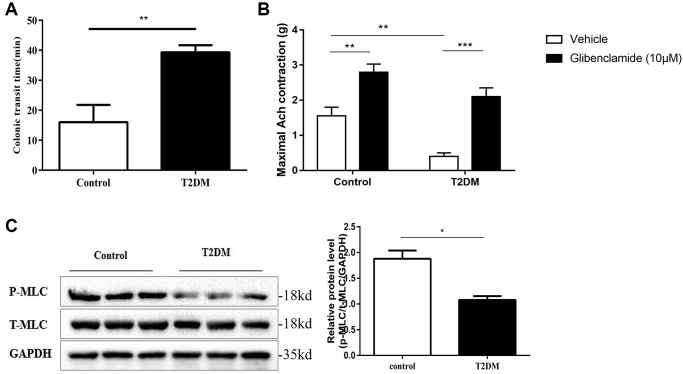
**Colonic motility dysfunction of T2DM rats.** (**A**) Distal colonic transit time at 8 w following an injection of STZ in the diabetic and control groups. (**B**) Distal colonic strips of diabetic and control rats were treated with vehicle (DMSO) or glibenclamide (10 μM). Quantification of maximum contractile force of a circular muscle strip stimulated by 10^−4^ mol/L ACh. (**C**) Western blotting analysis of the phosphorylation of MLC in the colon muscle layers. Relative expression was normalized to that of GAPDH. Left panel: Representative image; right panel: Quantitative analysis from three independent experiments. ^*^P < 0.05, ^**^P < 0.01, ^***^P < 0.001, control group *n* = 10, T2DM group *n* = 8.

Compared with the controls, the distal colon ejection response time was increased in the T2DM rats. (*P* < 0.01, Figure 2A). Muscle strip contractility assays showed that ACh (10^−4^ mol/L) increased the tension of distal colonic strips in both the normal and T2DM rats. The maximum contractile tension was increased about twice under the treatment of glibenclamide in the normal group, while it increased to about 5 fold in the T2DM group (*P* < 0.01, Figure 2B). Western blotting demonstrated that MLC phosphorylation, which is a prerequisite for smooth muscle contraction, was decreased significantly in colonic muscle tissues of T2DM rats (*P* < 0.05, Figure 2C). All these results indicated colonic motility dysfunction in T2DM rats.

### VF significantly inhibited the contractility of colonic smooth muscle strips from normal SD rats

**Figure 3:**
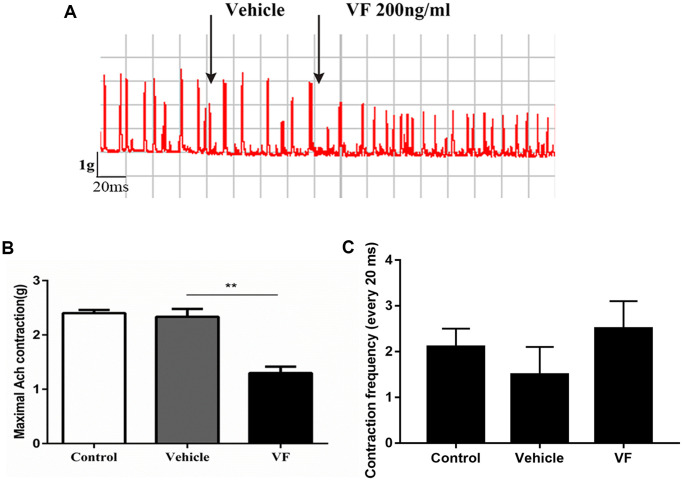
**VF significantly inhibited the contractility of colonic smooth muscle strips from normal SD rats.** Distal colonic strips of normal rats were treated with vehicle (PBS solution) or VF (200 ng/mL). Circular muscle strip contractility was recorded. (**A**) Representative image. (**B**) Quantitative analysis of contractile force. (**C**) Quantitative analysis of contractile frequency. ^*^P < 0.05, *n* = 6.

An *in vitro* study of distal colonic strips recorded the contractility of the colon (Figure 3). There was no change when the vehicle was added to the Krebs solution; however, the contractile tension decreased significantly after VF (200 ng/ml) stimulation. (*P* < 0.01, *n* = 6), suggesting that VF impaired colonic contractility.

### The expression of K_ATP_ channels were altered in T2DM rats

**Figure 4:**
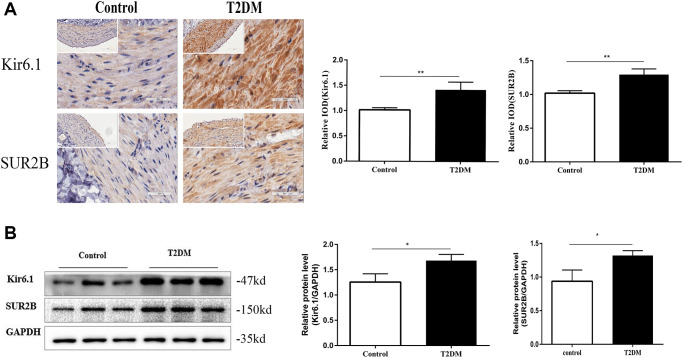
**The expression of K_ATP_ channels were altered in T2DM rats.** (**A**) Immunohistochemical staining of Kir6.1 and SUR2B subunits of K_ATP_ channels in the colon muscle layers in the two groups of rats at 18 w. Left panel: Representative image; right panel: Quantitative analysis. Low magnification, 200×, scale bar = 100 μm; high magnification, 400×, scale bar = 50 μm. (**B**) Western blotting analysis of Kir6.1 and SUR2B subunits of K_ATP_ channels in the colon muscle layers in the two groups of rats at 18 w. Relative expression was normalized to that of GAPDH. Left panel: Representative image; right panel: Quantitative analysis. ^*^P < 0.05, ^**^P < 0.01, control group *n* = 10, T2DM group *n* = 8.

Immunofluorescence staining of distal colonic sections showed that levels of Kir6.1 and SUR2B subunits of K_ATP_ channels were increased in the muscle layer in T2DM rats compared with that in the controls (both *P* < 0.01, Figure 4A). Western blotting revealed that the protein levels of Kir6.1 and SUR2B in the colonic smooth muscle of T2DM rats were increased considerably (both *P* < 0.01, Figure 4B). Thus, colonic K_ATP_ expression was increased in T2DM rats.

### VF reduced MLC phosphorylation and intracellular Ca^2+^ concentration in isolated colonic smooth muscle cells

**Figure 5:**
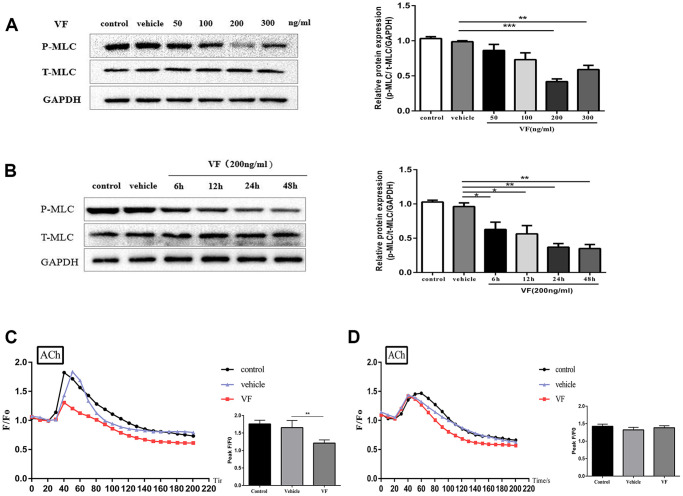
**VF reduced MLC phosphorylation and intracellular Ca^2+^ concentration in isolated colonic smooth muscle cells.** (**A**) CSMCs were treated with vehicle (PBS solution) or 0, 50, 100, 200, and 300 ng/mL VF for 24 h. Western blotting analysis of the phosphorylation of MLC in CSMCs stimulated by 0.1 μM ACh for 30 s. Relative expression was normalized to that of GAPDH. Left panel: Representative image; right panel: Quantitative analysis from three independent experiments. (**B**) CSMCs were treated with vehicle (PBS solution) or 200 ng/mL VF for 0–48 h. Western blotting analysis of the phosphorylation of MLC in SMCs stimulated by 0.1 μM Ach for 30 s. Relative expression was normalized to that of GAPDH. Left panel: Representative image; right panel: Quantitative analysis from three independent experiments. (**C**) Changes in fluorescence intensity caused by [Ca^2+^]_i_ relative to baseline (F/F0) in CSMCs treated with vehicle (PBS solution) or 200 ng/mL VF for 24 h. F0 was derived from the averaged intensity of the first 0–30 seconds. CSMCs were incubated in regular HBSS base buffer. Left panel: Fluorescence intensity of [Ca^2+^]_i_; right panel: Quantitative analysis of peak F/F0 and a representative image of fluorescence at peak F/F0 from three independent experiments (three cells in each experiment). (**D**) Changes in fluorescence intensity caused by [Ca^2+^]_i_ relative to baseline (F/F0) in CSMCs treated with vehicle (PBS solution) or 200 ng/mL VF for 24 h. F0 was derived from the averaged intensity of the first 0–30 s. CMSCs were incubated in calcium-free HBSS base buffer. Left panel: Fluorescence intensity of [Ca^2+^]_i_; right panel: Quantitative analysis of peak F/F0 and a representative image of fluorescence at peak F/F0 from three independent experiments. ^**^P < 0.01, ^***^P < 0.001.

Western blotting demonstrated significantly reduced levels of phosphorylated MLC in the 200 ng/mL VF group (*P* < 0.01, Figure 5A); 200 ng/mL was considered the most effective VF concentration and was used in subsequent experiments. The cells were treated with 200 ng/mL VF for 6–48 h, and the maximal effect was observed at 24 and 48 h (both *P* < 0.001; Figure 5B. The difference between the two groups was not statistically significant (*P* > 0.05), so 24 hours was chosen as the optimal intervention time.

The effect of VF on Ca^2+^ mobilization *in vitro* was then investigated. Laser confocal microscopy showed no significant difference in the increase in intracellular Ca^2+^ concentration between the normal control group and the vehicle control group after ACh stimulation (*P* > 0.05, Figure 5C); in comparison, the increase in intracellular Ca^2+^ concentration in the VF group was significantly lower (*P* < 0.01, Figure 5C), suggesting that VF could inhibit the increase of intracellular Ca^2+^ concentration in CSMCs. The effect of VF on the different components of Ca^2+^ mobilization was investigated further by resuspending the CSMCs in calcium-free HBSS base buffer. In the absence of extracellular Ca^2+^, all increases in intracellular Ca^2+^ would represent the release of Ca^2+^ from intracellular stores only. Under these conditions, there was no difference in the increase in intracellular Ca^2+^ concentration between the VF and control groups (*P* > 0.05, Figure 5D), suggesting that VF has no significant effect on the release of Ca^2+^ from intracellular calcium pools; thus, VF may mainly suppress the ACh-induced increase of Ca^2+^ concentration in CSMCs by reducing Ca^2+^ influx.

### Altered VF expression induced Kir6.1 upregulation

**Figure 6:**
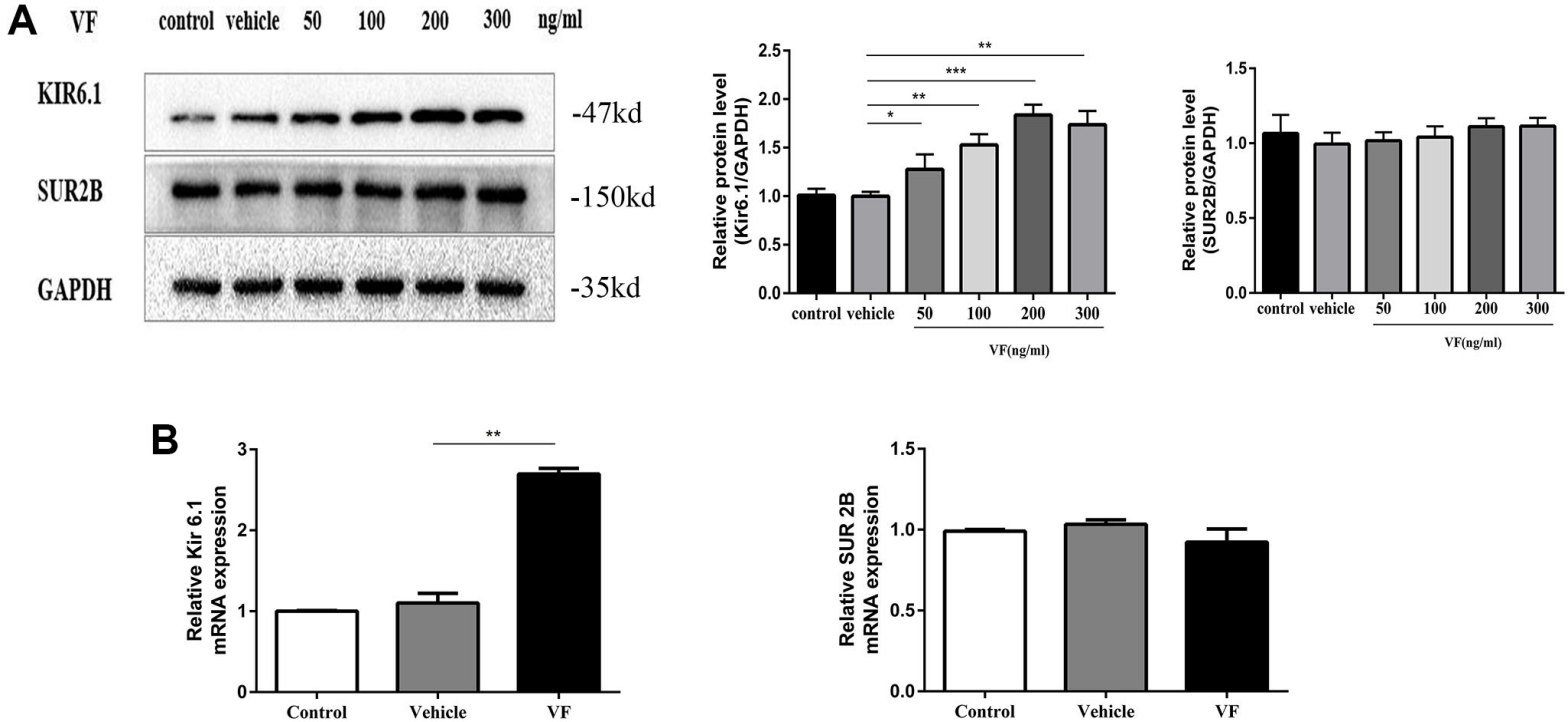
**Altered VF expression induced Kir6.1 upregulation.** (**A**) CSMCs were treated with vehicle (PBS solution) or 0, 50, 100, 200, and 300 ng/mL VF for 24 h. Western blotting analysis of K_ATP_ subunit protein levels in all the groups. Relative expression was normalized to that of GAPDH. Left panel: Representative image; right panel: Quantitative analysis from three independent experiments. (**B**) qRT-PCR analysis of K_ATP_ subunit mRNA levels in CSMCs treated with vehicle (PBS solution) or 200 ng/mL VF for 24 h. Relative expression was normalized to that of GAPDH. Left panel: Kir6.1 subunit; right panel: SUR 2B subunit. ^**^P < 0.01, ^***^P < 0.001.

We next examined whether VF increases K_ATP_ levels in CSMCs. The expression of the Kir6.1 subunit increased significantly when treated with 200 ng/mL VF for 24 h (*P* < 0.001, Figure 6A). qRT-PCR also showed that the expression of the Kir6.1 subunit mRNA in the VF group was significantly higher than that in the vehicle group (*P* < 0.01, Figure 6B). However, VF did not influence the expression of the SUR2B subunit. Therefore, we focused on the Kir6.1 subunit.

### Overexpression of Kir6.1 decreases intracellular Ca^2+^ concentration ([Ca^2+^]_i_) and MLC phosphorylation

The Kir6.1 subunit was overexpressed a using plasmid to identify the relationship between Kir6.1 and intracellular Ca^2+^ mobilization and MLC phosphorylation in CSMCs.

**Figure 7:**
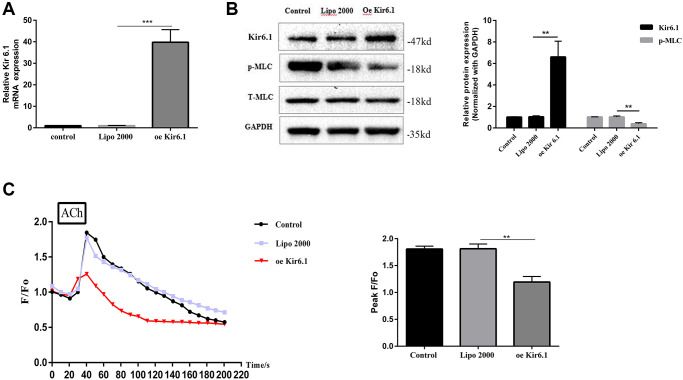
**Overexpression of the Kir6.1 subunit decreases intracellular Ca^2+^ concentration ([Ca^2+^]_i_) and MLC phosphorylation.** (**A**) qRT-PCR analysis of Kir6.1 subunit mRNA levels in CSMCs transfected with blank or Kir6.1 expression plasmids. Relative expression was normalized to that of GAPDH. (**B**) Western blotting analysis of Kir6.1 subunit protein levels and phosphorylation of MLC in CSMCs transfected with blank or Kir6.1 expression plasmids. Relative expression was normalized to that of GAPDH. Left panel: Representative image; right panel: Quantitative analysis from three independent experiments. (**C**) Changes in fluorescence intensity caused by [Ca^2+^]_i_ relative to baseline (F/F0) in CSMCs transfected with blank or Kir6.1 expression plasmids. F0 was derived from the averaged intensity of the first 0–30 s. CMSCs were incubated in calcium-free HBSS base buffer. Left panel: Fluorescence intensity of [Ca^2+^]_i_; right panel: Quantitative analysis of peak F/F0 and a representative image of fluorescence at peak F/F0 from three independent experiments. ^**^P < 0.01, ^***^P < 0.001.

Compared with that in the control group, the level of Kir6.1 mRNA of the Kir6.1 overexpression group increased significantly, indicating successful transfection (*P* < 0.001, Figure 7A). The intracellular p-MLC level and Ca^2+^ concentration of the Kir6.1 overexpression group were significantly reduced compared with those in the control group (both *P* < 0.01, Figure 7B–7C). This suggested that Kir6.1 exerts negative feedback on Ca^2+^ signals and MLC Phosphorylation in CSMCs. VF intervention increased the expression of Kir6.1 in CSMCs, indicating that VF might be involved in the contractile dysfunction of CSMCs by upregulating Kir6.1 levels.

### Kir6.1 upregulation was induced via the ROS/NF-κB-mediated pathway

**Figure 8:**
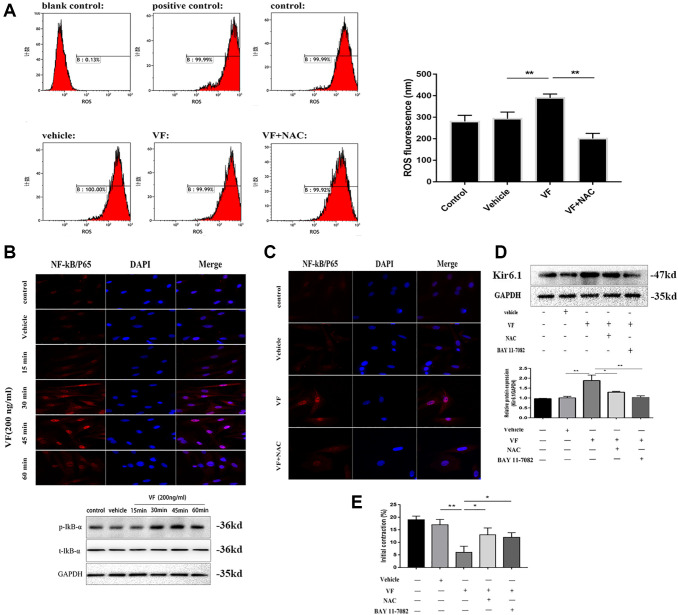
**Kir6.1 upregulation was induced via the ROS/NF-κB-mediated pathway.** (**A**) Flow cytometry analysis of the levels of ROS in CSMCs treated with vehicle (PBS solution) or VF (200 ng/mL), with or without NAC (1 mmol/L) for 24 h. Left panel: Representative image; right panel: Quantitative analysis from three independent experiments. Positive control: Rosup, 50mg/ml. Control: No treatment. (**B**) Upper panel: Representative double immunofluorescence staining of NF-κB/P65 activity in CSMCs treated with vehicle (PBS solution) or VF (200 ng/L) for 0–60 min. Nuclei were identified by DAPI staining. Lower panel: Western blotting analysis of phosphorylated IκB-αin CSMCs treated with vehicle (PBS solution) or VF (200 ng/L) for 0–60 min. (**C**) Representative double immunofluorescence staining of NF-κB/P65 activity in CSMCs treated with vehicle (PBS solution) or VF (200 ng/L), with or without NAC (N-acetyl-L-cysteine, 1 mmol/L), for 1 hour. Nuclei were identified by DAPI staining. (**D**) Western blotting analysis of Kir6.1 subunit protein in CSMCs treated with vehicle (PBS solution) or VF (200 ng/L), with or without NAC (1 mmol/L) or BAY 11-7082 (10 mg/mL), for 1 hour. Upper panel: Representative image; Lower panel: Quantitative analysis from three independent experiments. (**E**) Measurement of contraction of CSMCs treated with vehicle (PBS solution) or VF (200 ng/L), with or without NAC (1 mmol/L) or BAY 11-7082 (10 mg/mL), for 45 min by scanning micrometry. ^*^P < 0.05, ^**^P < 0.01, ^***^P < 0.001.

Flow cytometry showed that ROS levels were increased significantly in VF-treated CSMCs (Figure 8A). The results showed that the levels of phosphorylated IκB-α and translocation of NF-κB/P65 from cytoplasm to nuclei were increased in CSMCs exposed to exogenous VF for different times (15, 30, 45, and 60 min), and the maximal effect was observed at 45 minutes (Figure 8B). Pretreatment with the ROS inhibitor NAC for 1 h reversed the VF-induced increase in NF-κB/P65 activity significantly (Figure 8C), suggesting that ROS act upstream of NF-κB/P65. In addition, Kir6.1 upregulation was reduced significantly by NAC pretreatment and NF-κB inhibitor BAY 11-7082 treatment, separately (both *P* < 0.01, Figure 8D). This suggested strongly that VF induces Kir6.1 upregulation in CSMCs through the ROS/NF-κB pathway. In addition, we found that the contraction of CSMCs in the present study refers to the initial peak contraction occurred at 60 upon the addition of Ach. As shown in Figure 8E, the initial contraction of CSMCs were decreased significantly under the treatment of VF. However, this can be reversed by NAC and BAY 11-7082 pretreatment, suggesting that VF decreased the contractility of smooth muscle cells through the ROS/NF-κB pathway.

## DISCUSSION

The present study focused on the role of VF and K_ATP_ channels (Kir6.1/SUR 2B) in the regulation of colonic smooth muscle contraction in T2DM rats. We found that in T2DM rats, the distal colon ejection response time was delayed significantly, the contractility of colonic smooth muscle strips was weakened, and MLC phosphorylation was decreased, indicating the occurrence of colonic motility dysfunction. Meanwhile, the expression of VF and K_ATP_ channels was significantly enhanced in the T2DM rats’ colons. *In vitro*, VF inhibited the contractility of colonic smooth muscle strips, increased the expression of the Kir6.1 subunit, and decreased intracellular Ca^2+^ concentration, thereby decreasing MLC phosphorylation and CSMC contraction. Furthermore, inhibition of ROS/NF-κB signaling reversed VF–induced upregulation of Kir6.1 expression. These results indicated that the VF-induced upregulation of Kir6.1 expression acted via the ROS/NF-κB pathway, and interfered with Ca^2+^ signaling, thereby contributing to colonic contractile dysfunction. Thus, VF might be involved in the development of diabetic colonic motility disorders.

Visfatin is a pro-inflammatory adipocytokine discovered in 2005 [21], comprising 473 amino acid residues with a relative molecular weight of 52 kDa [21]. VF levels are increased in pathological conditions such as diabetes and obesity, and VF mediates low-grade inflammation induced by metabolic disorders, leading to the development of T2DM and its complications [22]. VF’s nicotinic acid ribose transferase (NAMPT) activity is the rate-limiting step for Nicotinamide adenine dinucleotide (NAD+) synthesis, acting by regulating intracellular NAD+ concentrations, and causing ROS overload; VF mainly exerts its pro-inflammatory effects through this pathway [22–24]. NF-κB, p38MAPK, ERK1/2, and PI3K are VF’s major downstream signaling molecules [25, 26]. VF can inhibit uterine smooth muscle contraction, resulting in obesity/metabolic syndrome in women with weak uterine contractions, with a stronger effect than traditional adipokines such as leptin [27]. VF can also induce relaxation of aortic smooth muscle, which might be related to endothelial nitic oxide synthase [15]. However, no studies have shown an effect of VF on colonic smooth muscle. The results of the present study showed that VF expression in serum and CSM increased in T2DM rats. *In vitro* study, VF inhibited the contractility of colonic smooth muscle strips, and decreased MLC phosphorylation of MLC, which indicated that VF plays an important role in colon dysmotility in T2DM.

Smooth muscle contraction and diastolic activity are closely related to changes in intracellular Ca^2+^ concentration [28, 29]. Given the important role of Ca^2+^ in smooth muscle cell contraction, we explored the effect of VF on the Ca^2+^ concentration in CSMCs. Intracellular Ca^2+^ mainly comes from the release of intracellular calcium pools and extracellular Ca^2+^. Experiments using acetylcholine showed that VF inhibited the increase in Ca^2+^ in CSMCs mainly by reducing extracellular Ca^2+^ influx. CSMCs membrane depolarization generates action potentials and induces contraction. Therefore, it is possible to regulate conductance (such as non-selective cation channels) or hyperpolarized conductance (K^+^ conductance) such as by K_ATP_ channels, double-pore potassium channels, delayed rectification, and calcium-activated potassium by regulating the depolarization of gastrointestinal smooth muscle to regulate its electrophysiology and contraction [5, 30]. In this study, we used a “Ca^2+^-free buffer” to exclude a role for Ca^2+^ influx. This is not usually sufficient to completely exclude a contribution of Ca^2+^ influx to changes in intracellular Ca^2+^ unless a chelator is also included in the buffer. Activation of K_ATP_ channels cause smooth muscle cell membrane hyperpolarization, reduce voltage-dependent L-type Ca^2+^ channel calcium inflow, and inhibited contraction [30, 31]. A variety of adipokines are suggested to increase K_ATP_ channel expression or increase its activity [32]. We found that VF could increase the expression of the Kir6.1 subunit of K_ATP_ channels in CSMCs membranes at the protein and mRNA levels, but had no effect on SUR2B, indicating that K_ATP_ channels mediate the inhibition of colon smooth muscle contraction by VF.

The main binding site of the K_ATP_ channel opener is in the SUR subunit, and similar to Kir6.1-/-mice, Sur2b-/-mice also show spontaneous coronary spasm and hypertension, indicating that the SUR2B subunit also plays an important role in the regulation of vascular smooth muscle tone [33]. Our results showed that the SUR2B subunit was increased in the muscularis of the colon of T2DM rats; however, surprisingly, VF had no significant effect on the expression of SUR2B mRNA and protein. Similar changes in Kir6.1 and SUR2B subunits have been observed in other studies. In a lipopolysaccharide-induced experimental colitis model, qRT-PCR showed that Kir6.1 gene expression was increased in colon smooth muscle cells by almost 22-fold, while during inflammation, SUR2B was decreased by 3-fold [34]. Kir6.1 mRNA was increased after myocardial ischemia/reperfusion, while Kir6.2 and SUR2B mRNA levels remained unchanged [35]. These results suggested that the transcription of the Kir and SUR genes encoding K_ATP_ channel subunits might have different regulatory mechanisms. It is unclear how changes in the transcriptional regulation of the two subunits affect functional channel complexes; thus, further research is needed to clarify the mechanism of this differential regulation and its pathophysiological significance. Besides, up-regulation of K_ATP_ channels is not the sole cause of reduced colonic contractility in diabetic rats. Reduced colonic contractility may resulted from impaired neuronal conduction and decreased muscarinic receptor sensitivity [36], up-regulation of PDGFRα + cells and small-conductance Ca^2+^-activated potassium channels [37].

VF can exert its pro-inflammatory effects through various pathways, such as ROS/NF-κB, in various cells [25, 26], and activation of NF-κB can increase the expression of K_ATP_ channels [38, 39] Therefore, we speculated that VF might increase the expression of Kir6.1 in CSMCs through the ROS/NF-κB pathway. ROS contributes to the cascade of intracellular signaling related to the inflammatory response [40, 41]. Our results showed that 200 ng/mL VF intervention increased the ROS levels in colon smooth muscle cells. NF-κB transcription factors not only participate in gene regulation during various physiological and pathological processes, such as immune responses, virus replication, apoptosis, and proliferation, but also play key roles in gene regulation of the inflammatory response. In our study, VF treatment in CSMCs increased the level of p65 levels in the nucleus, indicating enhanced NF-κB activity. In addition, ROS can induce NF-κB activation by modifying the activity of one or more kinases in the NF-κB activation cascade [40, 41]. We found that VF-induced NF-κB activation was reduced in CSMCs after inhibiting ROS generation, suggesting that ROS is upstream of the NF-κB signaling pathway. Activation of NF-κB can increase K_ATP_ channel expression. NF-κB activation during sepsis is associated with upregulation of vascular K_ATP_ channel mRNA and protein levels [39]. Moreover, pretreatment with the ROS inhibitor NAC or the NF-κB inhibitor BAY 11-7082 attenuated the expression of Kir6.1 subunits significantly in VF-induced K_ATP_ channels, indicating that ROS/NF-κB signaling is required for Kir6.1 subunit upregulation in VF-induced K_ATP_ channels.

This study had some limitations. The role of ion channels is mainly affected by the number of channels and channel activity. In our study, because of the experimental conditions and experimental techniques, the detection of ion channel activity could not be performed, and only the expression of K_ATP_ channels was detected. Whether K_ATP_ channel activity is altered requires further verification. Second, our results provide associations but do not test the hypothesis by showing that knocking down visfatin signaling in diabetic rats prevents the observed changes in motility. Furthermore, we studied the effect of VF on distal colonic contraction in SD rats. Further studies can be performed to determine whether VF has the same effect on smooth muscle from different intestinal segments.

In conclusion, we found that VF might cause contractile dysfunction of CSMCs by upregulating the expression of the Kir6.1 subunit of K_ATP_ channels via the ROS/NF-κB pathway and by altering Ca^2+^ signaling. The VF levels in serum and colonic smooth muscles were elevated in T2DM; thus, VF might be involved in the development of diabetic colonic motility disorders.
